# Effect of Joule Heating on Resistive Switching Characteristic in AlO_x_ Cells Made by Thermal Oxidation Formation

**DOI:** 10.1186/s11671-019-3229-y

**Published:** 2020-01-15

**Authors:** Xinxin Zhang, Ling Xu, Hui Zhang, Jian Liu, Dingwen Tan, Liangliang Chen, Zhongyuan Ma, Wei Li

**Affiliations:** 1 School of Electronic Science and Engineering, Nanjing University, Nanjing, China; 2 Collaborative Innovation Center of Advanced Microstructures, Nanjing University, Nanjing, China

**Keywords:** Joule heating, AlO_x_-based RRAM, conductive filament, oxygen vacancies

## Abstract

The AlO_x_-based resistive switching memory device is fabricated by an oxidation diffusion process that involves depositing an Al film on an ITO substrate and annealing at 400 °C in a vacuum. An AlO_x_ interface layer with a thickness of ~ 20 nm is formed as a resistance switching layer. Bipolar and unipolar resistive switching (RS) behaviours are obtained when the compliance current is limited (≥ 1 mA). In the unipolar RS behaviour, the devices fail to perform set/reset cycles at a low temperature (40 K), which suggests that Joule heating is essential for the unipolar RS behaviour. In the bipolar RS behaviour, the abrupt reset transforms into a gradual reset with decreasing temperature, which suggests that Joule heating affects the rupture of the conductive filament. In addition, the conductive mechanisms in the high-resistance state and low-resistance state are revealed by the temperature dependence of the I-V curves. For the low-resistance state, the conduction mechanism is due to the electron hopping mechanism, with a hopping activation energy of 9.93 meV. For the high-resistance state, transport mechanism is dominated by the space-charge-limited conduction (SCLC) mechanism.

## Background

Resistive switching random access memory (RRAM) has attracted extensive attention as one of the most promising candidates for next-generation non-volatile memory [1–4]. Compared with traditional commercialized flash memory and other emerging non-volatile memories, the RRAM device has a simple structure (MIM), fast write/erase speed and excellent endurance and retention performance [5–8]. As one of the resistive switching materials compatible with conventional complementary metal-oxide semiconductor technology, AlO_x_-based RRAM has also been extensively studied, having more attractive application potential due to its multilevel storage capability and self-rectification [9, 10]. Generally, two switching types are observed in metal-oxide devices: (1) unipolar switching, which is not dependent on the polarity of the applied voltage and (2) bipolar switching, which relies on the polarity of the applied voltage. Their inherent switching mechanisms are different. Many factors can affect the type of resistive switching, such as the device structure, electrode materials and programming current [11]. The coexistence of unipolar and bipolar switching has been reported in some metal-oxide materials, such as HfO_2_, NiO and ZnO [12–16]. The bipolar resistive switching (RS) behaviour is related to the formation/rupture of conductive filaments composed of oxygen vacancies. The unipolar RS behaviour is often due to a thermal damage conductive filament or phase structure transition. The bipolar RS behaviour is usually observed in AlO_x_-based RRAM. The coexistence of unipolar and bipolar behaviours in AlO_x_ RRAM has rarely been reported, and the physical switching mechanism in the unipolar RS behaviour has still not been clarified.

In this paper, we report the coexistence of the unipolar and bipolar RS behaviours in AlO_x_-based RRAM. By studying the resistive switching characteristics of unipolar and bipolar switching for different compliance currents, Joule heating is used to explain the rupture conductive filaments in the reset process of the unipolar RS behaviour. When the local temperature inside conductive filaments reaches the critical temperature, the conductive filaments are broken, and the unipolar RS behaviour occurs. Moreover, the use of Joule heating to assist in rupturing conductive filaments in the reset process is proposed for the bipolar RS behaviour. The effect of Joule heating is well verified by placing the device at different temperatures. Meanwhile, the performance effect at different temperatures for AlO_x_ RRAM is also investigated. The stability and controllability of the RS behaviour is essential for applying RRAM arrays in the future. A deeper understanding of the effect of Joule heating in the resistive switching process is important and necessary. Moreover, we investigate the conductive mechanism by the temperature dependence of the current for high-resistance state (HRS) and low-resistance state (LRS).

## Methods

**Fig. 1:**
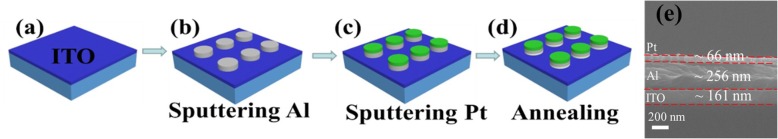
A schematic diagram of the fabrication process. (**a**) ITO/glass substrate. (**b**) Deposition of the Al electrode by sputtering. (**c**) Pt covering the Al electrode. (**d**) Formation of the AlO_x_ interface layer by annealing at 400 °C in a vacuum. (**e**) SEM image of the annealed Pt/Al/ITO device. The thicknesses of the Pt, Al and ITO are approximately 66 nm, 256 nm and 161 nm, respectively

The resistive switching memory devices based on AlO_x_ are fabricated by the following process. The schematic diagram is shown in Fig. 1(a)–(d). The Al and Pt are sputtered on the surface of the ITO glass substrate in sequence with a shadow mask to form circular spots with a 200-μm diameter. The Pt layer covering the Al can be used to avoid oxidation of the Al surface during the following annealing process. The device is annealed at 400 °C for 4 h in a vacuum. An unannealed sample is used for reference. The cross-section scanning electron microscope (SEM) photograph reveals the structure of the device. A three-layer structure of the annealed Pt/Al/ITO device is shown in the inset of Fig. 1(e). The top layer is a Pt electrode (~ 66 nm). The middle layer is an annealed Al layer (~ 256 nm). The bottom layer is an ITO electrode (~ 161 nm). The microstructure of the device is analysed by high-resolution transmission electron microscopy (HRTEM). The distribution of elements is obtained by using energy dispersive X-ray (EDX) spectroscopy on the same equipment. The I-V test is carried out using the Agilent B1500A semiconductor parameter analyser in dc sweep mode at room temperature. The temperature dependence of the I-V characteristic is detected in the Lake Shore CRX-4K system under a vacuum of 5 × 10^−5^ Torr.

## Results and Discussion

**Fig. 2:**
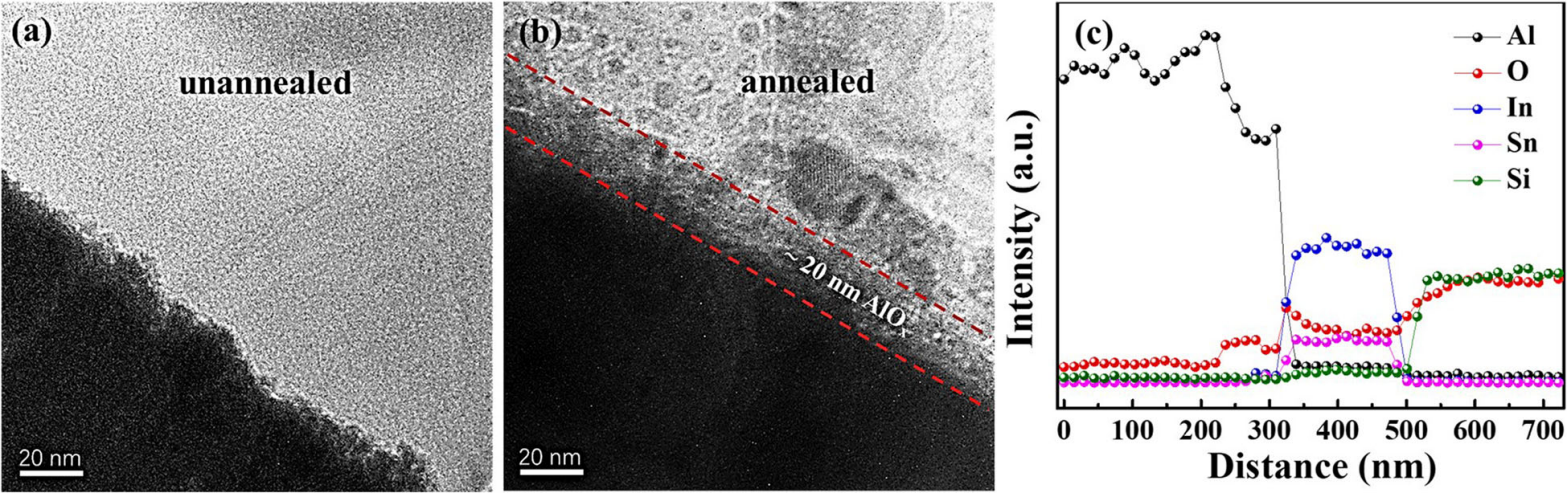
**a** Cross-section HRTEM image of the unannealed Pt/Al/ITO. **b** Cross-section HRTEM image of the annealed sample after 4 h. An interface layer is formed. **c** The energy dispersive X-ray (EDX) spectra of five elements (Al, O, In, Sn and Si)

To check the microstructure changes after annealing the Pt/Al/ITO devices, HRTEM is used to check the region between the Al and ITO glass substrates. Figures 2a and b show the unannealed and annealed samples, respectively. Compared to the unannealed sample, an obvious interface layer is found in the annealed sample after 4 h. The thickness of the interface layer is ~ 20 nm. The EDX spectra are used to identify the element distribution between Al and ITO, as shown in Fig. 2c. An obvious diffusion of oxygen atoms occurred at the interface of the Al/ITO interface during the annealing process. Other elements (In, Sn) do not display significant diffusion in the EDX spectra. Compared to other metals, Al has a lower standard Gibbs free energy (− 1582.9 KJ/mol) to form the corresponding metal oxides [17]. We infer that the interface AlO_x_ layer formed during the annealing process.

**Fig. 3:**
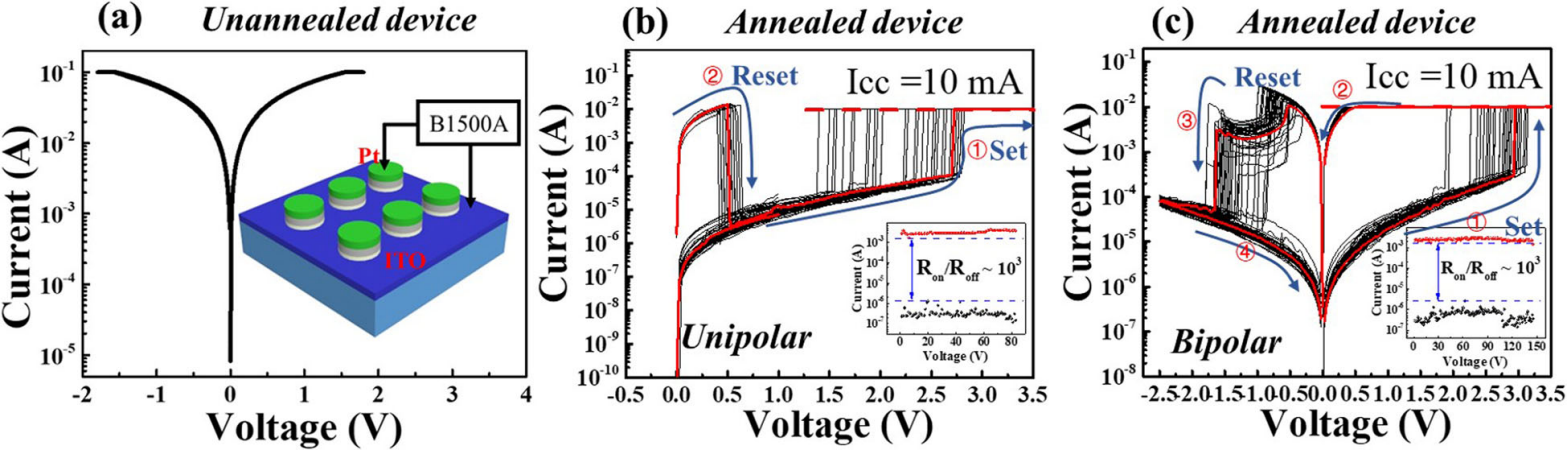
**a** The I-V curve for the unannealed Pt/Al/ITO device. The inset shows a schematic diagram of the electrical measurement. The Pt top electrode is the applied bias voltage, and the ITO is grounded. **b** The 50–cycle I-V curve for unipolar switching (annealed for 4 h). The dashed line denotes the compliance current Icc = 10 mA. The red line indicates the first set process and reset process. The arrows indicate the voltage sweeping direction. The read voltage is set to 0.1 V. The inset shows the endurance characteristic. **c** The 50-cycle I-V curve for bipolar switching (annealed for 4 h). The inset shows the endurance characteristics. The read voltage is set to 0.1 V

Figure 3a shows the unannealed sample current-voltage (I-V) characteristic. No resistive switching behaviour is observed, which is consistent with the unannealed TEM results. No AlO_x_ resistive switching layer is formed. The inset shows a schematic diagram of the electrical measurement. During the I-V measurement, the voltage is applied to the top electrode (Pt), and the bottom electrode (ITO) is grounded. The annealed devices are also measured under the same conditions. The annealed device shows the coexistence of the unipolar and bipolar RS behaviours. The two RS behaviours can be activated independently. Figure 3b shows 50-cycle sweep curves of the unipolar RS behaviour. The compliance current is set to 10 mA to avoid hard breakdown of devices during the set process. The arrows indicate the voltage sweeping direction. A positive voltage sweep (0 V → 3.5 V) is applied to the Pt electrode. The device switches from a high-resistance state to a low-resistance state (set process or programming process). Afterward, another voltage sweep (0 V → 1 V) causes an abrupt current reduction with the compliance current removed. The device switches to the HRS (reset process or erasing process). No obvious larger forming voltage is required to activate the device. The inset displays the 80-cycle endurance characteristics, and the ratio of *R*_on_/*R*_off_ is approximately 10^3^ using a read voltage of 0.1 V. Figure 3c shows the bipolar RS behaviour. The RS behaviour is observed in the opposite voltage polarity. The set and reset sweeping voltages follow the sequence of 0 V → +3.4 V → 0 V → − 2.5 V → 0 V. The device switches from the HRS to the LRS when a positive bias voltage is applied to the Pt top electrode. Then, it is switched back to the HRS under a negative bias voltage. Similar to the unipolar case, no obvious electroforming process is observed. The inset shows the endurance characteristics for 150 cycles. The ratio of *R*_on_/*R*_off_ is approximately 10^3^ using a read voltage of 0.1 V.

Generally, the bipolar RS behaviour is often observed in AlO_x_-based RRAM devices. The bipolar switching mechanism is due to forming/rupturing conductive filaments composed of oxygen vacancies [11, 16]. When a positive voltage is applied to the top electrode, the oxygen ions (O^2−^) migrate to the top electrode, leaving oxygen vacancies. Oxygen vacancies are accumulated to form the conductive filaments. The device switches to the LRS. When a negative voltage is applied to the top electrode, the oxygen ions are extracted back to AlO_x_ and the conductive filaments rupture. The bipolar switching mechanism is related to the electrochemical mechanism. However, the set process and reset process occur with the same voltage polarity for the unipolar switching behaviour. The unipolar resistive switching is triggered by conductive filament thermal breakdown. The switching mechanism is explained by a thermal-based mechanism in other RRAM devices [16]. To verify that the Joule heating accounts for the unipolar switching behaviour in AlO_x_ RRAM, a different compliance current is used to control the current flow through the device.

**Fig. 4:**
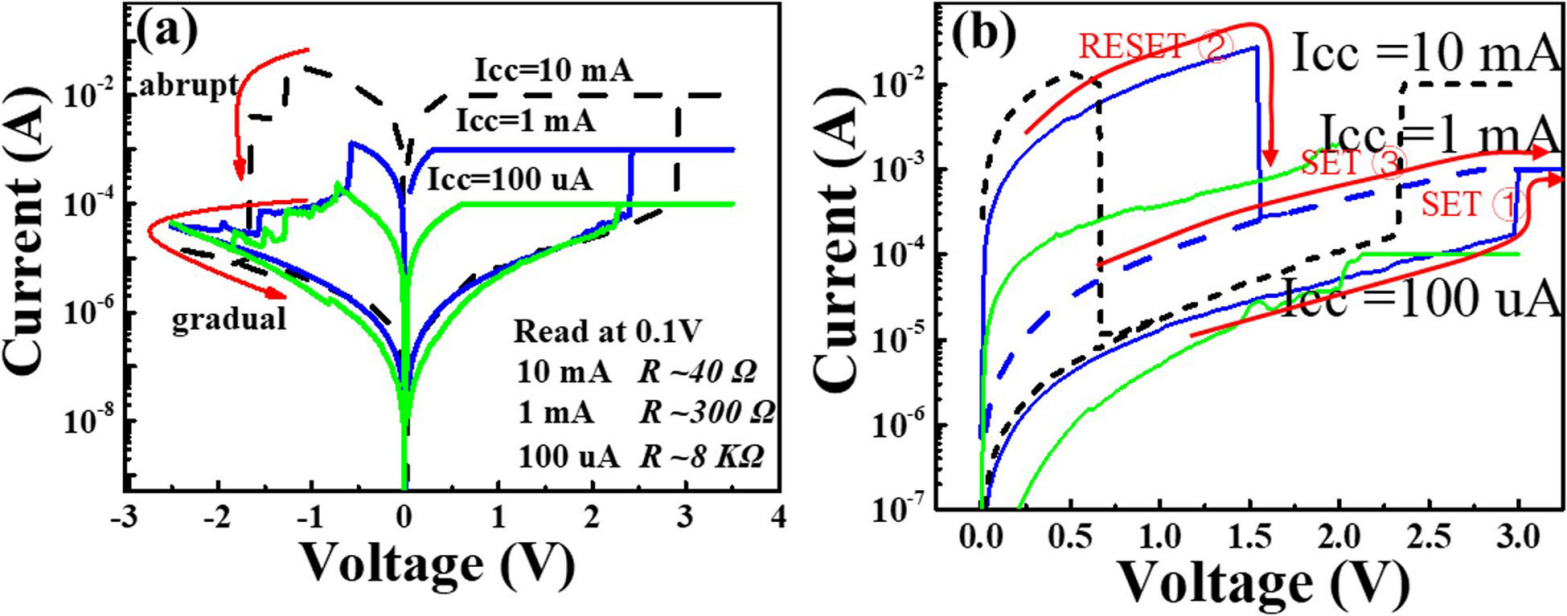
**a** The I-V curves of the bipolar at different compliance currents: Icc = 10 mA (dotted line), Icc = 1 mA (blue line) and Icc = 100 uA (green line). The LRS resistance at different compliance currents at read 0.1 V (Icc = 10 mA, RLRS ~ 40 Ω; Icc =1 mA, RLRS ~ 300 Ω; Icc =100 uA, RLRS ~ 8 KΩ). **b** The I-V curves of the unipolar behaviour at different compliance currents: Icc = 10 mA (dotted line), Icc = 1 mA (blue line) and Icc = 100 uA (black line)

Figure 4a shows the I-V characteristic of the bipolar switching behaviour for different compliance currents. The conductive filament resistance can be controlled by the setting compliance current. A lower resistance of the LRS (Icc = 10 mA, R_LRS_ ~ 40 Ω; Icc =1 mA, R_LRS_ ~ 300 Ω; Icc = 100 uA, R_LRS_ ~ 8 KΩ) can be obtained by increasing the compliance current. The resistance in the LRS (*R*_LRS_) varies from tens of ohms to thousands of ohms under different compliance currents. The different *R*_LRS_ values are related to the formation of different conductive filament sizes under different compliance currents. Joule heating decreases with decreasing filament size [18]. Notably, when the compliance current Icc = 100 uA and Icc = 1 mA, a gradual reset process is observed during the reset process in the bipolar RS behaviour, which is different from the abrupt reset at Icc = 10 mA. The gradual reset is explained by the progressive rupture of the conductive filament [19]. The abrupt reset is related to Joule heating assistance rupture [20]. The influence of Joule heating on the bipolar RS behaviour is reflected in the abrupt reset process. The bipolar RS behaviour can be considered a combination of an electrochemical mechanism and Joule heating at high programming currents [13, 21].

Figure 4b shows the unipolar characteristics under different compliance currents (Icc = 10 mA, Icc = 1 mA and Icc = 100 uA). The unipolar switching is observed only at a compliance current Icc = 10 mA and 1 mA. Compared to the reset voltage of the compliance current Icc = 10 mA within 1 V, the reset voltage (Icc = 1 mA) is obviously increased over 1.5 V and the reset current decreases by approximately two orders of magnitude (~ 724 uA) after the reset operation. The current value after the reset process approximates the compliance current. The device cannot reset to the initial state (~ 100 KΩ). Russo et al. proposed the critical temperature (*T*_crit_) for the unipolar reset process in the self-accelerated thermal dissolution model [22]. When the temperature inside the conductive filament reaches the critical value under an applied reset voltage between the two electrodes, the conductive filament is dissolved and broken in the reset state. The function relationship among the critical temperature, voltage, current and resistance can be described as follows:

$$ {T}_{\mathrm{crit}}={T}_0+{P}_{\mathrm{reset}}\cdotp {R}_{\mathrm{th}} $$

*T*_0_ is room temperature, *R*_th_ is the effective thermal resistance of the conductive filament, which has a weaker size dependence, and the electric power can be written as*P*_reset_ = *V*_reset_ · *I*_reset_. For the lower compliance current Icc = 1 mA, a larger reset voltage is needed. When the hottest point of the conductive filament reaches the critical temperature, the thermal stability of the conductive filament worsens. The conductive filaments subsequently rupture. The unipolar RS behaviour then occurs. However, the LRS current is smaller for compliance current Icc = 100 uA. Even if the reset voltage increases, the current value faces difficulty in reaching the current level at the larger compliance current (Icc = 1 mA and Icc = 10 mA). The Joule heating generated is not sufficient to reach the critical temperature. Thus, no unipolar RS behaviour is observed. If the reset voltage is increased further, the device may break. Therefore, the unipolar RS behaviour is driven by Joule heating in AlO_x_ RRAM.

**Fig. 5:**
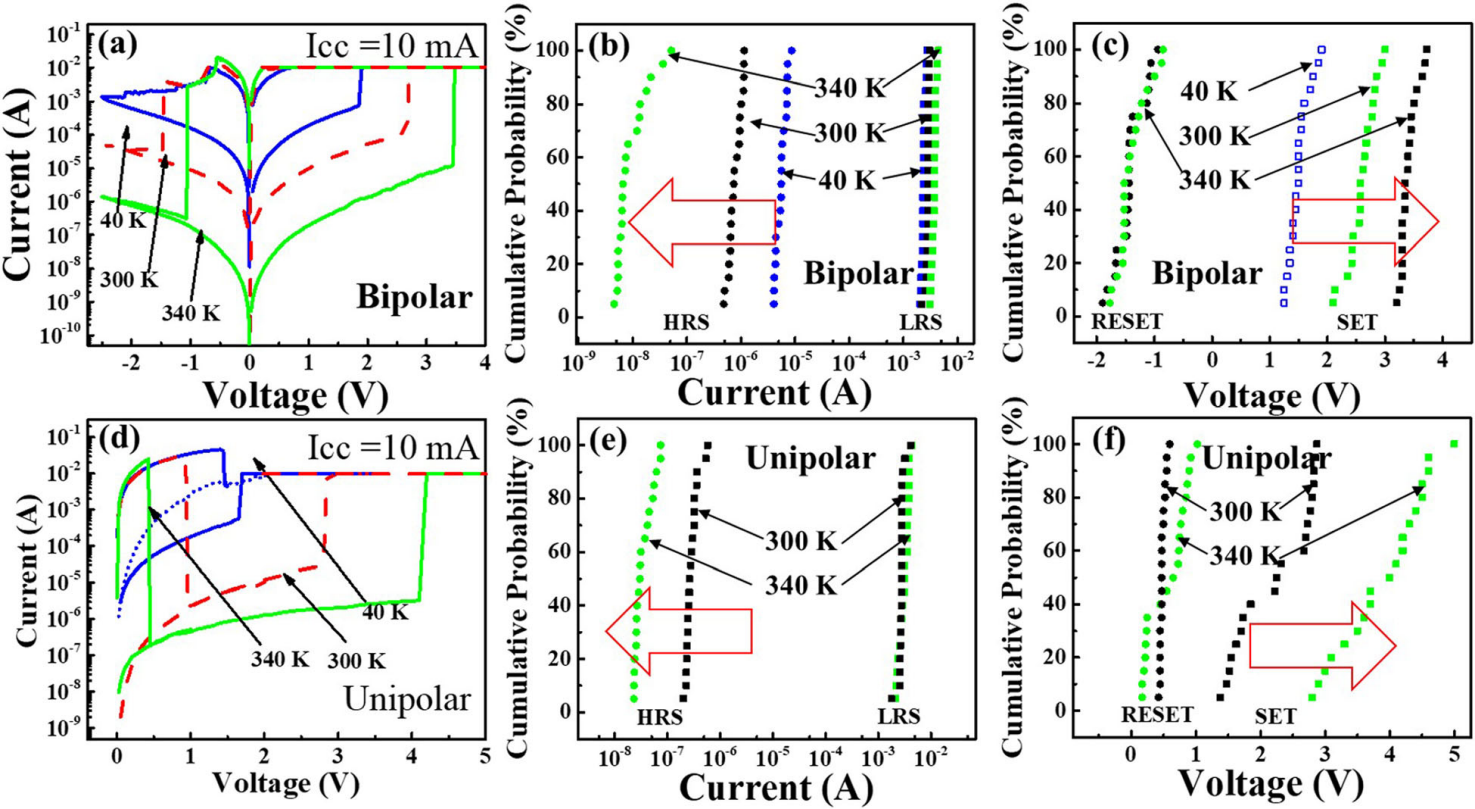
**a** The I-V curves of the bipolar behaviour at different temperatures (40 K (blue line), 300 K (red dashed line) and 340 K (green line)) with a compliance current Icc = 10 mA. **b** Statistical result of the HRS and LRS current for 20 bipolar switching cycles at different temperatures (40 K, 300 K and 340 K). **c** Statistical result of the SET and RESET voltage for 20 bipolar switching cycles at different temperatures (40 K, 300 K and 340 K). **d** The I-V curves of the unipolar behaviour at different temperatures (40 K (blue line), 300 K (red dashed line) and 340 K (green line)) with a compliance current Icc = 10 mA. The blue dotted line indicates the next set process after the reset operation. **e** Statistical result of the HRS and LRS current for 20 unipolar switching cycles at different temperatures (300 K and 340 K). **f** Statistical result of the SET and RESET voltage for 20 unipolar switching cycles at different temperatures (300 K and 340 K)

For further research on the influence of Joule heating on the RS behaviour, the devices are placed at different temperatures. During the set process, the compliance current Icc = 10 mA is used. The I-V curves of the bipolar behaviour are shown in Fig. 5a. It is worth noting that the abrupt reset process transforms into a gradual reset process with decreasing temperature down to 40 K. Compared to 300 K and 340 K, the Joule heating can be well dispersed at 40 K. The effect of the Joule heating can be reduced to a minimum. Thus, the electrochemical mechanism plays a major role during the reset process in the bipolar switching behaviour. The gradual reset process is explained by a partially ruptured conductive filament. The device cannot be reset to the initial state at the same reset voltage. This phenomenon is also observed in other metal-oxide materials [23]. Figures 5b and c show the statistical distribution of the operating current (HRS, LRS) and voltage (SET, RESET) in bipolar switching at different temperatures. Clearly, the HRS current decreases with increasing temperature. In addition, the SET voltage increases with increasing temperature. These observations suggest that the Joule heating affects the breakage of the conductive filaments. When the temperature is raised, fewer conductive filaments remain in the AlO_x_ resistive switching layer during the reset process. More insulating high-resistance states are obtained. The SET voltage obviously increases. The LRS current slightly increases with increasing temperature, which corresponds to the characteristic transport of a semiconductor. Figure 5d shows the I-V characteristic of the unipolar behaviour at different temperatures. Compared to 300 K and 340 K, the device cannot reset to the initial state at 40 K, which is due to thermal dissipation. The temperature inside the conductive filament does not reach the critical temperature. The conductive filament cannot be completely ruptured. The device cannot switch to the LRS again at the compliance current Icc = 10 mA (the blue dotted line). Figures 5e and f show the statistical distribution of the operating current (HRS, LRS) and voltage (SET, RESET) under unipolar switching at different temperatures. Similarly, a higher HRS current and a larger SET voltage with increasing temperature are observed. Thus, Joule heating is considered essential for the unipolar RS behaviour.

**Fig. 6:**
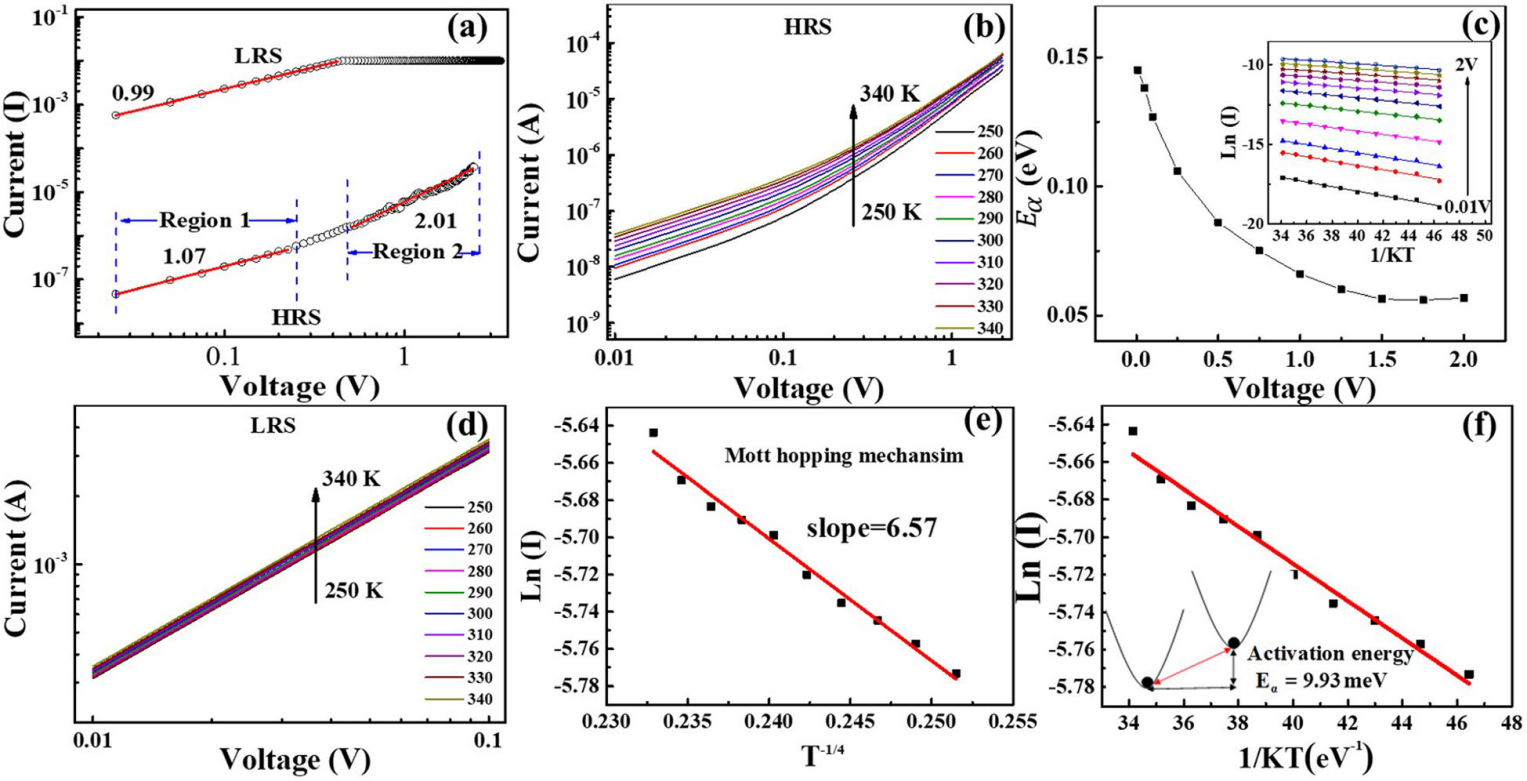
**a** Linear fitting for the I-V curves using a log-log scale in the positive bias. **b** Temperature dependence of the current for the HRS from 250 K to 340 K. **c** The activation energy *E*_*α*_ at different voltages is summarized. The inset shows an Arrhenius plot of the current temperature data at different voltages in the HRS. **d** Temperature dependence of the current for the LRS from 250 K to 340 K. **e** The relationship of conductivity ln I versus temperature T^−1/4^. The read voltage is 0.1 V. **f** The activation energy *E*_*α*_ = 9.93 meV is calculated

For a better study of the conduction mechanism, we preliminarily estimate the switching mechanism by fitting the I-V curve. The I-V curve is re-plotted in a double-logarithmic plot, as shown in Fig. 6a. The LRS shows an Ohmic conducting behaviour with a slope close to 1, which is probably caused by the formation of conductive filaments [24]. The HRS can be divided into two regions: in the low-voltage area (< 0.4 V, region 1), the Ohmic conduction behaviour is observed, whereas in the high-voltage area (> 0.4 V, region 2), the slope is close to 2. The transport behaviour is consistent with the space-charge-limited conduction (SCLC) [25]. In the SCLC model, the current density *J* for trap-controlled SCLC emissions can be described as

$$ {J}_{\mathrm{ohm}}=q{n}_0\mu \frac{V}{d} $$

$$ J=\frac{9}{8}{\varepsilon}_r{\varepsilon}_0\mu \theta \left(\frac{V^2}{d^3}\right) $$

where *q* is the elementary charge, *n*_0_ is the thermally generated free carriers, *μ* is the electron mobility, *ε*_*r*_ is the static dielectric constant, *ε*_0_ is the permittivity of the space, *θ* is the ratio of the free carrier density to total carrier density, *V* is the applied voltage and *d* is the film thickness. In region 1 (low applied voltage), corresponding to Ohm’s law (*I* ∝ *V*^1^), a small number of carriers may be generated due to thermal excitation and excited to the conduction band from the valence band or the impurity level in this region. When the applied voltage increases, the injected carriers become trapped. The conduction becomes space-charge-limited. The current of the HRS follows a square law (*I* ∝ *V*^2^) in region 2. Figure 6b shows the temperature dependence of the HRS current. The current increases with increasing temperature, which suggests a semiconductor-like conducting behaviour [26, 27]. From the slopes of Arrhenius-type plots of the data (the inset of Fig. 6c), the activation energy (*E*_*α*_) from 0.01 V to 2 V is as summarized in Fig. 6c. The results indicate that *E*_*α*_ is relatively high (~ 0.15 eV) in the low-voltage region and shows Ohmic conduction behaviour. As the voltage increases, *E*_*α*_ decreases, which is a characteristic feature of SCLC [28]. The temperature-dependent I-V analyses clearly support the SCLC conduction mechanism in the HRS.

Figure 6d shows that the current of the LRS slightly increases with increasing temperature, showing a semiconductor-like conducting behaviour. Metal conductive filaments are excluded. Figure 6e shows a linear relationship between ln (I) and T^−1/4^, which suggests that the mechanism of the LRS obeys Mott’s variable range hopping model [29, 30]. If the energy levels of two localized states are close enough and the wave functions overlap, electrons can hop between the two sites, assisted by thermal energy. The value of activation energy *E*_*α*_ is 9.93 meV for the LRS, as shown in Fig. 6f, which is smaller than 26 meV (the activation energy at room temperature). This value ensures the variable range hopping of the electrons at room temperature. In other metal-oxide semiconductors, the hopping mechanism is also observed in the LRS, and the I-V curve fitting shows the Ohmic conductive behaviour at room temperature [31]. Thus, the resistive switching mechanism in the LRS is related to oxygen vacancies in the conductive filaments.

**Fig. 7:**
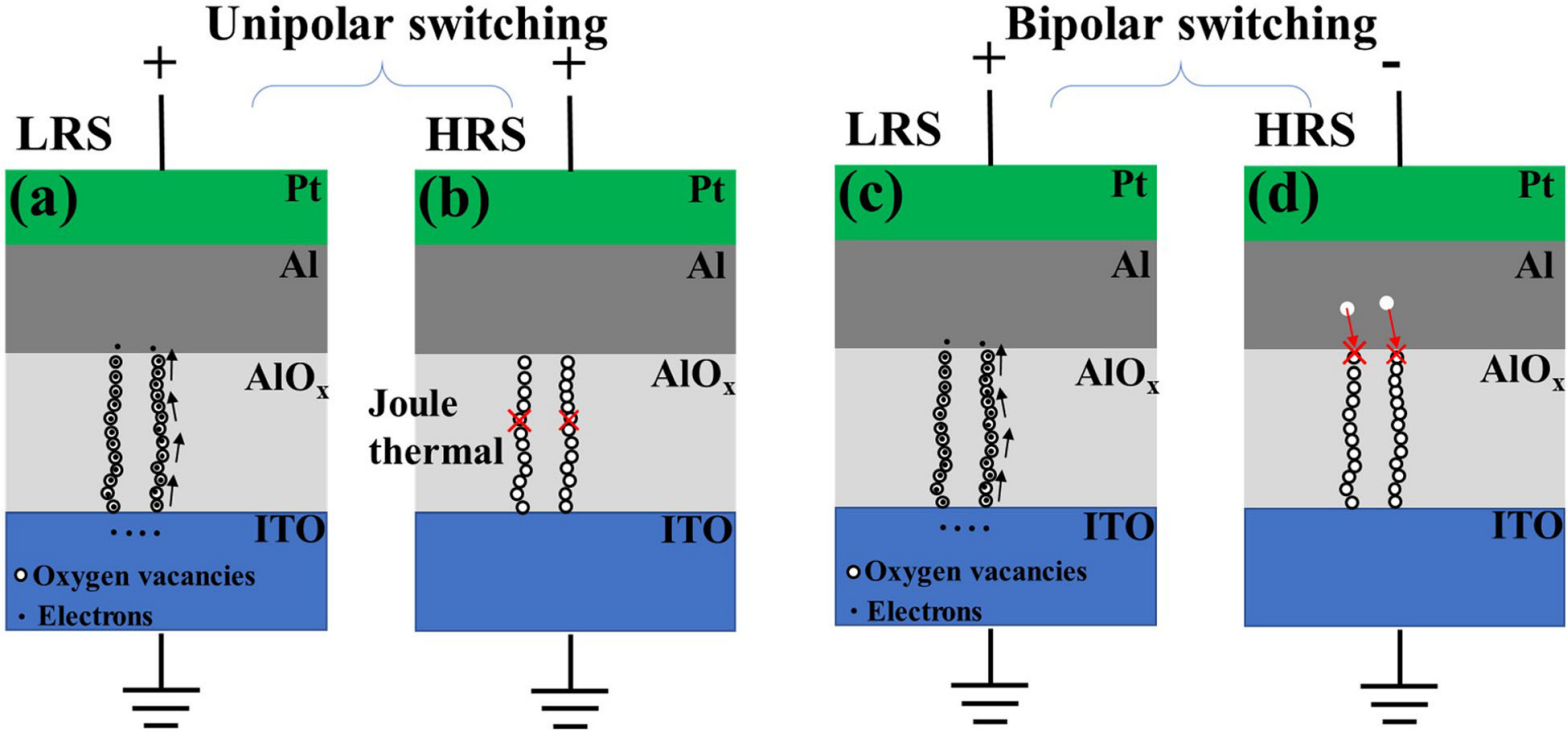
The switching mechanism schematic of the AlO_x_-based RRAM device. (**a**) Set process for unipolar switching under a positive voltage. Conductive filaments consist of oxygen vacancies. The black arrows indicate the electron migration direction. (**b**) Reset process for unipolar switching under a positive voltage. The conductive filament is ruptured by Joule heating. Electrons are trapped by defects. The conductive mechanism in the HRS is dominated by SCLC. (**c**) Set process for bipolar switching under a positive voltage. (**d**) Reset process for bipolar switching under a negative voltage. The conductive filaments rupture

Figure 7 illustrates the unipolar and bipolar resistive switching models. For the unipolar and bipolar RS behaviours in the set process, oxygen ions migrate towards the top electrode under an electric field. Finally, the oxygen ions are reduced, leaving oxygen vacancies in the AlO_x_ resistive switching layer. A large accumulation of oxygen vacancies forms oxygen conductive filaments between the ITO and unoxidized Al layers. The device is set to the LRS. The electrons hop through the conductive filament composed of oxygen vacancies, as shown in Fig. 7(a) and (c). For the unipolar RS behaviour in the reset process, the compliance current is removed. The positive bias is applied again, and the current increases with increasing voltage. When the highest temperature point inside the conductive filament reaches the critical temperature, the stability of the conductive filament becomes worse and is easily broken. The device switches to the HRS after the conductive filament is destroyed, as shown in Fig. 7(b). In the bipolar RS behaviour, a negative bias is applied to the top electrode. Oxygen ions are extracted back to the AlO_x_ interface layer. The conductive filaments rupture, as shown in Fig. 7(d). The device is reset to the HRS. When the reset current is relatively larger, Joule heating enhances the conductive filament rupture process. An abrupt transition in the reset process appears. The electron transport mechanism in the HRS is dominated by the SCLC mechanism in both RS behaviours.

## Conclusions

In this paper, the coexistence of the unipolar and bipolar resistive switching behaviours is observed in AlO_x_-based RRAM. By researching the current-voltage characteristics of the unipolar and bipolar switching at different compliance currents and varying work temperatures, we propose that Joule heating is essential for the unipolar resistive switching behaviour in AlO_x_-based RRAM. When a high programming current flows through the conductive filament in the reset process, the local temperature in the conductive filaments reaches the critical temperature, and the conductive filaments rupture. Unipolar RS behaviour occurs. In the bipolar resistive switching behaviour, the reset process is attributed not only to the electrochemical mechanism but also to the Joule heating. Thermal prompts rupture of the conductive filament when the device has a high erase current, which results in higher resistance of the HRS and a larger SET operating voltage in AlO_x_-based RRAM. Thus, Joule heating is a non-negligible factor of the RS performance. These results will help us deeply understand the influence of Joule heating on the resistive switching behaviour in AlO_x_-based RRAM. Furthermore, the conductive mechanism is studied. The conductive mechanism for the LRS is due to the electrons hopping through conductive paths. For the HRS, the conductive mechanism is dominated by the SCLC mechanism.

## Data Availability

All data and materials are available without restriction.

## Abbreviations

RS: Resistive switching
SCLC: Space-charge-limited conduction
RRAM: Resistive switching random access memory
HRS: High-resistance state
LRS: Low-resistance state
SEM: Scanning electron microscope
HRTEM: High-resolution transmission electron microscopy
EDX: Energy dispersive X-ray spectroscopy
